# Cruciform DNA in mouse growing oocytes: Its dynamics and its relationship with DNA transcription

**DOI:** 10.1371/journal.pone.0240844

**Published:** 2020-10-20

**Authors:** Xie Feng, Feng-Yun Xie, Xiang-Hong Ou, Jun-Yu Ma

**Affiliations:** 1 The Second School of Clinical Medicine, Southern Medical University, Guangzhou, China; 2 Fertility Preservation Lab, Reproductive Medicine Center, Guangdong Second Provincial General Hospital, Guangzhou, China; 3 Bioland Laboratory (Guangzhou Regenerative Medicine and Health Guangdong Laboratory), Guangzhou, China; Nanjing Agricultural University, CHINA

## Abstract

Cruciform DNA is a causing factor of genome instability and chromosomal translocation, however, most studies about cruciform DNA in mammalian cells were based on palindromic sequences containing plasmids and reports about endogenous cruciform DNA are rare. In this study we observed the dynamics of endogenous cruciform DNA in mouse growing oocytes using immunofluorescence labeling method. We found cruciform DNA foci exist in transcription active growing oocytes but not in transcription inactive fully grown oocytes and colocalized with Parp1 but not with DNA damage marker γH2A.X. By analyzing the Genotype-Tissue Expression data, we found cruciform DNA-mediated chromosomal translocation in human spermatocytes is associated with the specific DNA transcription in testis. When inhibiting the transcription with α-amanitin in mouse oocytes, we found oocyte cruciform DNA foci decreased significantly. In summary, we observed the endogenous cruciform DNA in growing oocytes and our results showed that the cruciform DNA formation is transcription-dependent.

## Introduction

When double-strand DNA which contain directed repeats are denatured, the intra-strand indirect repeats in both strands can anneal and form stem or stem-loop secondary structures. Then the stems or stem-loops with the up- and down-stream double-strand DNA will form a new specific four-way junction structure in DNA called cruciform DNA [1]. The formation of cruciform DNA, or cruciform extrusion, is mainly caused by the negative supercoiling in DNA [1]. Evidences indicated that cruciform DNA is a causing factor of genome instability and it can increase the risk of specific recurrent chromosomal translocation in human genome [2–5]. In these cruciform DNA-mediated translocations, t(11;22)(q23;q11) has the most higher prevalence in human and got most attentions and investigations [3]. Although t(11;22)(q23;q11) carriers are generally phenotypically normal, their fertility is lower comparing to normal individuals as chromosomal translocations induce miscarriage during pregnancy, and they also have higher risks of breast cancer [6]. In addition, offspring of t(11;22)(q23;q11) carriers might inherit unbalanced form of chromosomal translocation which could induce the Emanuel syndrome [7]. By amplifying the t(11;22) joint region in human tissues and cells by PCR, the de novo t(11;22)(q23;q11) translocations are found frequently in spermatocytes but not in other tissues of normal individuals [3,8,9] and the formation of t(11;22) (q23;q11) in spermatocytes is age- independent [10,11]. In addition to translocation, cruciform DNA can also induce genomic structural variants or isochromosomes in human genomes [12–15]. As cruciform DNA is a critical factor inducing genome instability, so that analyzing the dynamics of cruciform DNA would be helpful for us to further analyze how cruciform DNA mediate the DNA damage and then design methods to prevent the cruciform DNA-mediated chromosomal aberrations.

Although cruciform DNA is a common structure in genomic DNA and causing factor for human diseases, which factors promote cruciform DNA formation and how cruciform DNA induce genome stability are still not well understood. On the one hand, the formation of cruciform DNA induced DNA double-strand breaks (DSBs) might be cell-specific and be an instantaneous event, so we still don’t know at which time and in which cell the cruciform DNA is formed and in what conditions the cruciform DNAs could be transformed into DSBs [8]. On the other hand, there is few cell lines or animal models can be used to analyze the endogenous cruciform DNA formation and DSB transformation. Generally, only plasmids which contain palindromic sequences are used to analyze the stability of cruciform DNA in cells [2,16,17], however, the plasmid system lacks the genomic context and can’t fully mimic the intra-nuclear conditions.

Unlike somatic cell and spermatocyte, mammalian growing stage oocyte has relatively larger nucleus, which is termed as germinal vesicle [18] and convenient for observing the dynamics of nucleoplasmic signals. The growth of mammalian oocytes would generally take several days or several months in a wave by wave manner depending on different species [19–21], so it is possible for us to collect different stage oocytes from adult ovaries. For female mouse, when their oocytes are fully grown, the oocyte transcription activities will be silenced and a Hoechst positive ring-like structure will be formed Surrounding the Nucleolus [22]. These fully grown oocytes are called Surrounded Nucleolus (SN) oocytes and the growing but not fully grown oocytes are then named as Non-Surrounded Nucleolus (NSN) oocytes because there is no Hoechst positive ring-like structure surrounding the nucleolus [22]. In this study, we analyzed the dynamics of cruciform DNA in mouse oocytes. We found cruciform DNA in oocytes could be detected by immunofluorescence labeling using antibodies and its formation is associated with the DNA transcription. Our results showed that mouse oocytes could be used as a model to analyze the genome instability induced by cruciform DNA.

## Material and methods

### Oocyte collection and culturation

All animal manipulations in this study were performed according to the animal experiment standards and guidelines of Guangdong Second Provincial General Hospital and approved by the Ethical Committee of Guangdong Second Provincial General Hospital. All oocytes manipulations in this study were under the oocyte manipulation media M2 (Sigma, M7167) with 2.5 μM milrinone (MCE, HY-14252) to block the oocytes from meiosis resumption. To isolate the large antral follicle oocytes, ovaries of 18 days post partum (dpp) or 8-weeks old ICR strain female mice were firstly collected. Then ovaries were chopped with blade and oocytes were isolated with mouth pipette. To isolate the pre-antral follicle oocytes, we collected pre-antral follicles from chopped mouse ovaries and treated the follicles with Trypsin-EDTA solution (Solarbio, T1300) at 37°C for 25 minutes and removed the somatic cells by mouth pipette.

### Oocyte treatments

To inhibit the RNA polymerase II mediated transcription in oocytes, oocytes were treated with 25 μg/mL α-amanitin (MCE, HY-19610) for 6 hours. If the oocytes were used for labeling the newly synthesized RNAs, the oocytes were treated with 0.5 mM 5-ethynyl uridine (EU) (Click Chemistry Tools, 1261–10) for a further 2 hours.

### Immunofluorescence labeling and 5-ethynyl uridine click reaction

The antibodies used in this study are: cruciform DNA antibody [2D3] (GeneTex, GTX54648); γH2A.X antibody (Bioworld, BS4760); and Parp1 antibody (Sangon, D261071). Oocytes were firstly fixed with 4% Paraformaldehyde Fix Solution (Sangon, E672002) at room temperature (RT) for 20 minutes and permeated with 0.3% Triton at RT for 20 minutes. Then oocytes were treated with Quick Antigen Retrieval Solution for Frozen Sections (Beyotime, P0090) at RT for 45 minutes. After washing 2 times, oocytes were blocked with 1% BSA for 1 hour and incubated with primary antibodies at 4°C overnight. After washing 3 times, oocytes were incubated with secondary antibodies and then stained with Hoechst and mounted on glass slides. Then oocytes were observed under the Dragonfly Confocal system.

If EU click reaction was needed, we shorted the fixation and permeation time to 15 minutes and the antigen retrieval time to 10 minutes. After washing of secondary antibodies, we preformed the EU click reaction according the manual of BeyoClick EdU Cell Proliferation Kit with Alexa Fluor 647 (Beyotime, C0081S) but changed the reagent EdU with EU.

### Transcriptome data analysis

The transcriptome data used in this study are originated from the Genotype-Tissue Expression (GTEx) project [23] and downloaded from the UCSC genome browser [24]. The version of human genome assembly used in this study is GRCh38/hg38.

### Statistics

Students’ t test was used in this study to assess the significance of the foci number differences in oocytes. Fisher’s exact test was used to assess the significance of the oocyte proportion of different groups. P-value < 0.01 was marked by **.

## Results

### The dynamics of cruciform DNA in mouse oocytes

**Fig 1:**
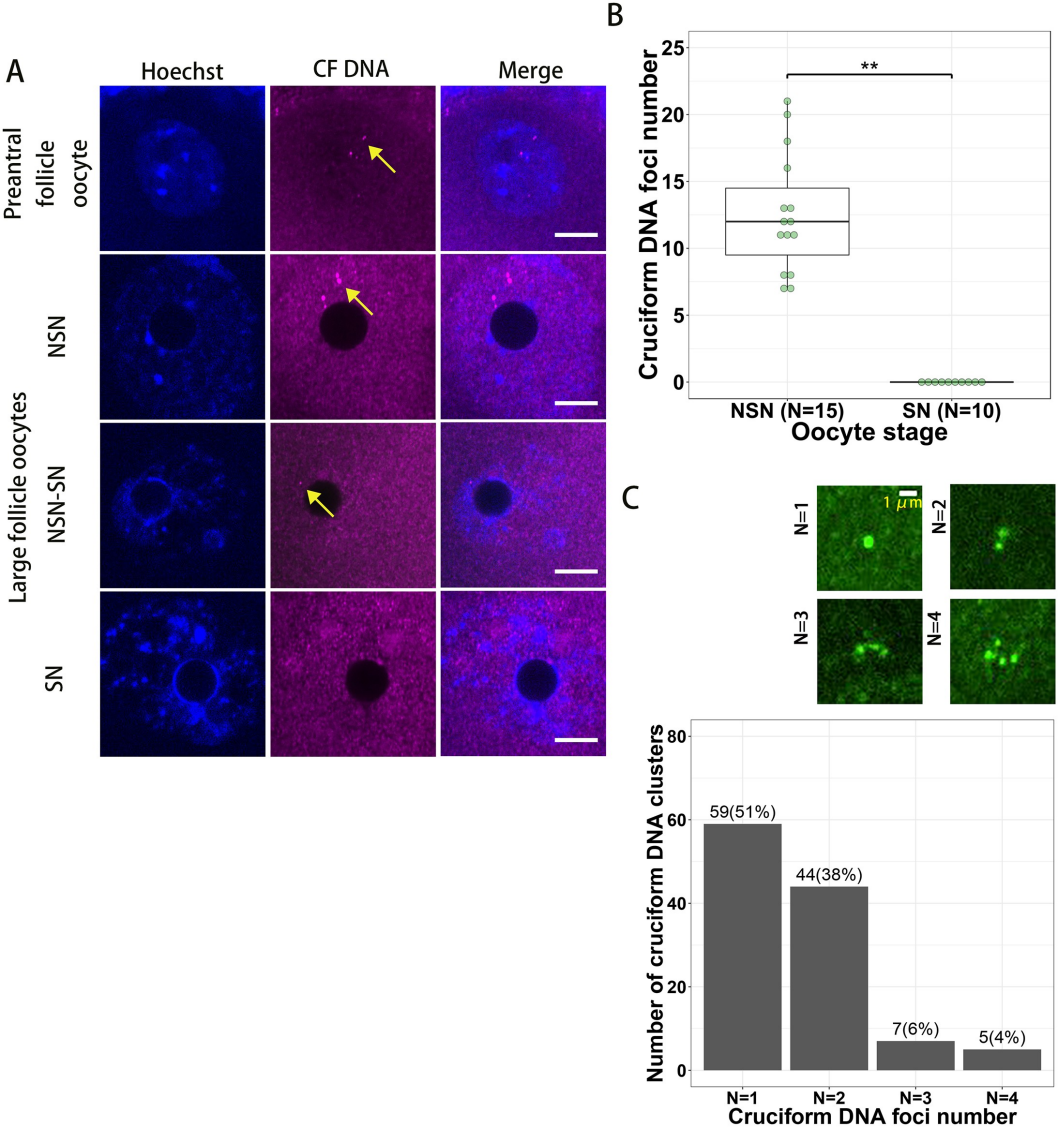
The dynamics of cruciform DNA foci in mouse oocytes. (**A**) Cruciform DNA foci (yellow arrows) can be detected in growing oocytes (preantral follicle oocytes, large antral follicle NSN and NSN-SN oocytes) but not in fully grown oocytes (SN oocytes). Bar = 10 μm. (**B**) The average number of cruciform DNA foci decreased from 12.5 in NSN oocytes to 0 in SN oocytes. (**C**) The ratio of cruciform DNA clusters with different number of cruciform DNA foci. CF, cruciform.

To analyze the localization and dynamics of cruciform DNA in mouse growing oocyte, we collected different stage oocytes from preantral follicles and large antral follicles of 8 weeks old mice. By immunofluorescence labeling of the cruciform DNA with antibodies we found that cruciform DNA could be detected in preantral follicle oocytes, NSN and NSN-SN (the transition stage of oocytes from growing NSN state to fully grown SN state) stage oocytes but not in the fully grown SN oocytes (Fig 1A). The average cruciform DNA foci number in NSN oocytes was 12.5 but when oocytes developed to the chromatin-condensed SN stage we couldn’t detected any cruciform DNA focus in normal oocytes (Fig 1B). For growing oocytes, every oocyte contains four copy of genomes (two sets of homologous chromosomes (2N) or four sets of chromatids (4C)) and the sister chromatids are hold together by cohesins and homologous chromosomes are linked with each other by chiasmates [25]. So it is not surprising that we could found the cruciform DNA foci formed clusters in oocytes (Fig 1C). In one cruciform DNA cluster, we could find 1–4 cruciform DNA foci, and clusters with one or two cruciform DNA foci were most common in oocytes (51% clusters contain one focus and 38% clusters contain two foci, Fig 1C).

### Oocyte cruciform DNA are colocalized with Parp1

**Fig 2:**
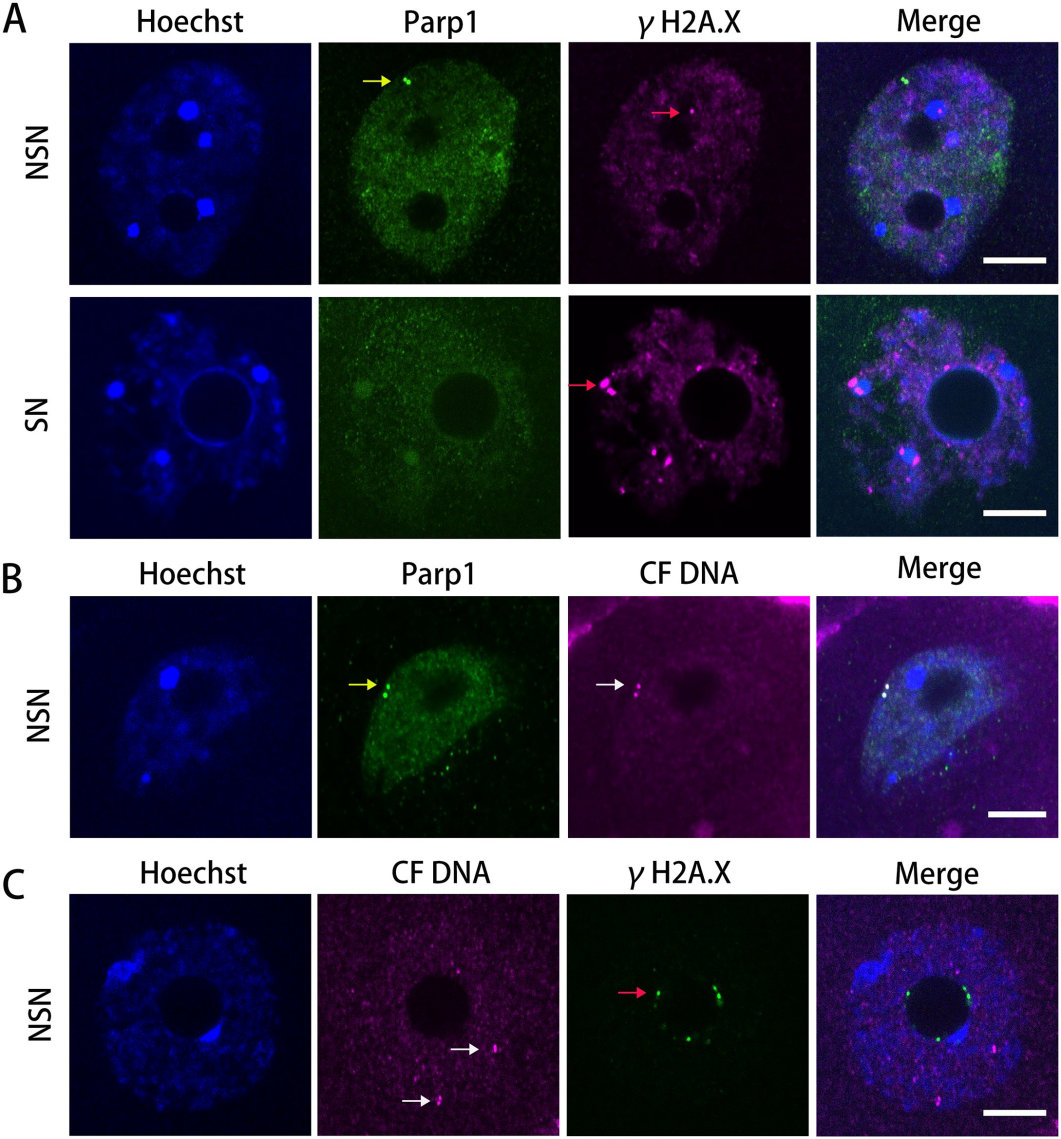
Cruciform DNA foci in oocytes are colocalized with Parp1. (**A**) Parp1 formed foci (yellow arrows) in NSN oocytes but not in SN oocytes and they are not colocalized with γH2A.X (red arrows). (**B**) Parp1 colocalized with cruciform DNA foci (white arrows) in oocytes. (**C**) Cruciform DNAs are not colocalized with γH2A.X in oocytes. CF, cruciform. Bar = 10 μm.

Evidences had suggested that Parp1 could bind at the ends of hairpin arms of cruciform DNA on plasmids [26]. In this study, to verify whether Parp1 binds to endogenous cruciform DNA, we labeled Parp1 in oocytes with antibodies. We found that Parp1 could be detected in the NSN oocytes but not in the SN oocytes and didn’t colocalize with endogenous DNA damage regions (labeled by γH2A.X) in oocytes [27] (Fig 2A). Just like cruciform DNA, the Parp1 foci could also form clusters in NSN oocytes (Fig 2A). As expected, when we labeled both Parp1 and cruciform DNA in NSN oocytes, we found Parp1 foci colocalized with the cruciform DNA foci (Fig 2B). In addition, we also found cruciform DNA were not colocalizd with γH2A.X in oocytes (Fig 2C), indicating there is rare cruciform DNA induced DSB in normal oocytes.

### The formation of cruciform DNA is associated with transcription

**Fig 3:**
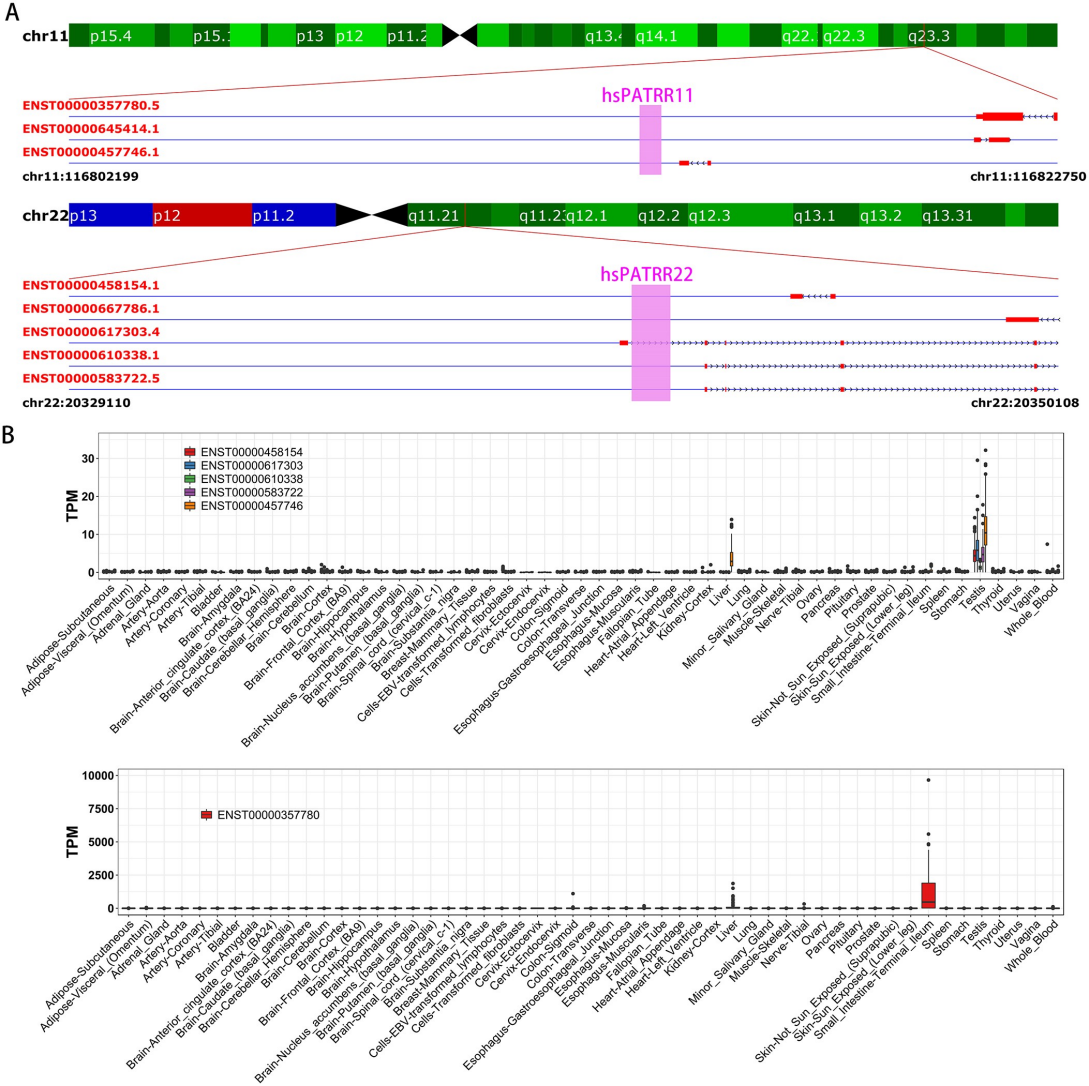
Genome stabilities of PATRR11 and PATRR22 regions in human are associated with transcription. (**A**) The location of PATRR11 and PATRR22 (marked by pink rectangle) on chr11 and chr22, and their associated transcripts. (**B**) The expression patterns of PATRR adjacent transcripts in human tissues.

As DNA transcription is the main cause factor of negative supercoiling in DNA [28], we next analyzed the association between DNA transcription and the formation of cruciform DNA. As the palindromic AT-rich repeats (PATRRs) regions on human chr11 and chr22 are fragile sites to form cruciform DNA-mediated chromosomal translocation in spermatocytes [3,8], so we analyzed the association between PATRR regions and DNA transcription. We analyzed the transcription patterns of PATRR11 and PATRR22 associated transcripts in different human tissues using the GTEx dataset [23]. As a result we found both PATRR11 and PATRR22 regions are associated with testis specific transcribed transcripts (Fig 3). For the PATRR11, its nearest transcript ENST00000457746 (~1 kb downstream of PATRR11) is specifically expressed in liver and testis (Fig 3B). However, the transcript ENST00000357780 located ~8 kb away from PATRR11 don’t show testis specific expression pattern (Fig 3B). Human PATRR22 located at the intron region of ENST00000617303, and we found ENST00000617303 was also a testis specifically expressed transcript (Fig 3). These results indicated that the cruciform mediated genome instability at PATRR11 and PATRR22 regions in spermatocytes might be resulted from that the PATRR11 and PATRR22 regions are transcriptionally active in spermatocytes.

**Fig 4:**
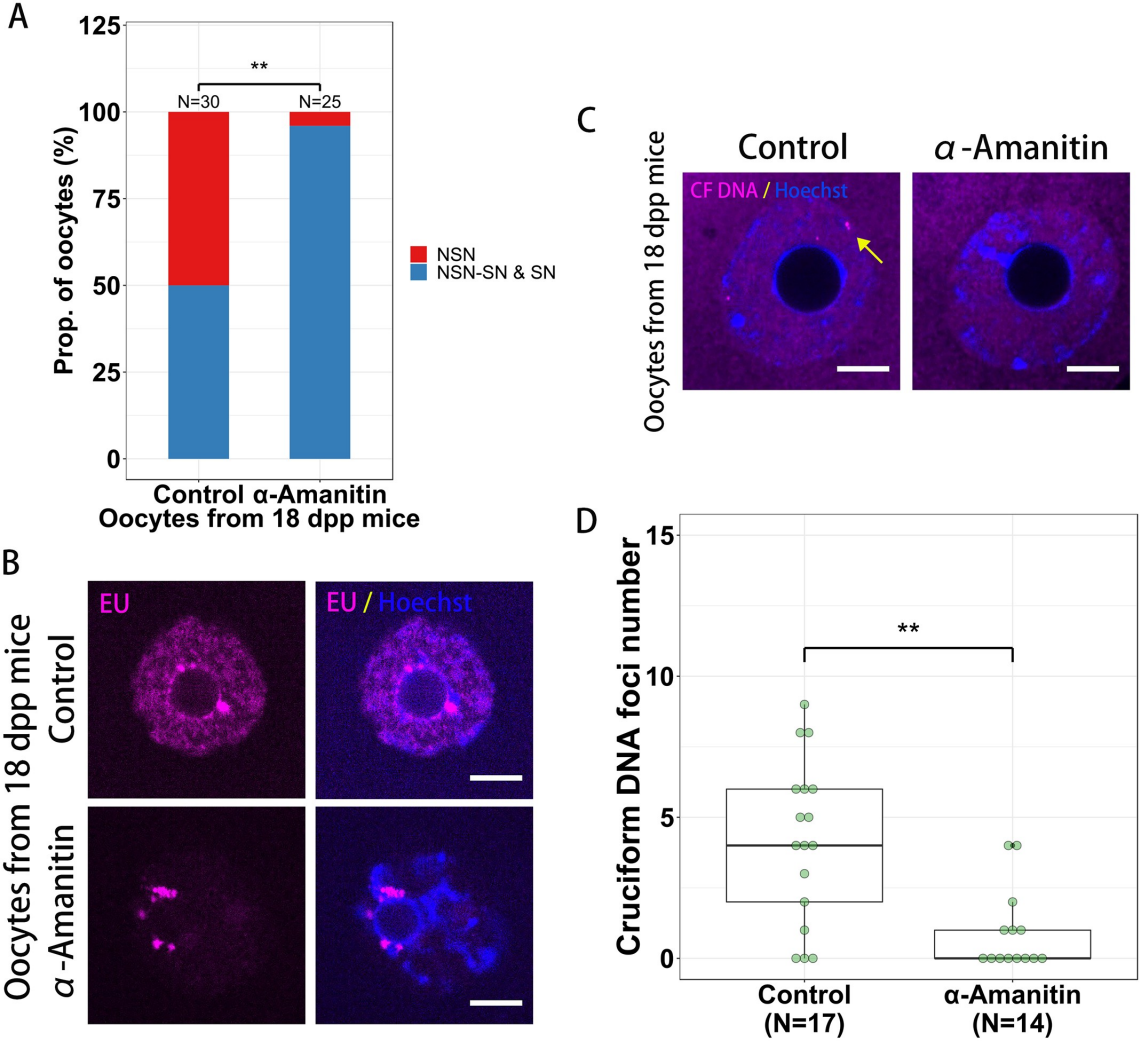
The formation of cruciform DNA in oocytes is transcription dependent. (**A**) RNA polymerase II inhibitor α-amanitin induces oocyte chromatin configuration changed from less condensed NSN or NSN-SN state into more condensed state. (**B**) RNA polymerase II based transcription is inhibited but RNA polymerase II non-dependent transcription is still active in α-amanitin-treated oocytes. (**C** and **D**) The cruciform DNA foci (yellow arrows) numbers are decreased in α-amanitin-treated oocytes. CF, cruciform. Dpp, days post partum. Bar = 10 μm.

In addition to the analysis of GTEx data, we also analyzed the effects of DNA transcription on the cruciform DNA formation in oocytes. We chose 18 dpp old mouse oocytes for this analysis as most of these oocytes are either NSN oocytes or NSN-SN oocytes which are transcription active, cruciform DNA foci positive and easier for us to do statistical analysis. When we treated the 18 dpp mouse oocytes with RNA polymerase II inhibitor α-amanitin [29–31], we found the condensed chromatin configurations of oocytes were increased (Fig 4A). After labeling the newly synthesized RNA by EU click reaction, we found the global transcription in oocytes was inhibited by α-amanitin, but some sites around nucleolus still be transcriptionally active which might be transcribed by RNA polymerase I or III and couldn’t be inhibited by α-amanitin (Fig 4B). As a result, we found the cruciform DNA foci were significantly decreased in α-amanitin-treated oocytes (p < 0.01, Fig 4C and 4D), indicating the formation of cruciform DNA in growing oocytes was also transcription-dependent.

## Discussion

In this study, we analyzed the dynamics of endogenous cruciform DNA in growing oocytes. As we known, a single cruciform DNA molecule is hard to observe under confocal microscope, so we think the bright cruciform DNA foci detected by antibodies might be tandem cruciform DNA structures but not be only one single cruciform DNA structure. Indeed, in mammalian genome, there are lots of microsatellites which are AT-rich [32] and these AT-rich microsatellites might form tandem cruciform DNA structures in oocytes. However, we still don’t know the precise positions of cruciform DNA foci in mouse genome, it is needed to be deep analyzed.

As cruciform DNA will increase the risk of genomic instability, so cells need to resolve the hairpin structures on cruciform DNA to decrease the risk of DNA damage. However, how the hairpin structures on cruciform DNA are resolved by cells were still not well known. Using palindromic sequences containing plasmids, structure specific DNA endonucleases had been found being essential for cells to resolve the cruciform DNAs [2]. In our results we had found the decrease of cruciform DNA foci number from NSN to SN oocytes, however, we hadn’t found the colocalization of γH2A.X with cruciform DNA foci. These results suggested the resolving of cruciform DNA might not induce DNA breaks in normal oocytes.

From our results we also found the formation of cruciform DNA is strongly associated with DNA transcription. Our results indicated that spermatocyte specific transcription of adjacent regions of palindromic sequences (such as PATRR11 and PATRR22) might be the causing factor of the cruciform DNA-mediated genomic instability. This result also suggested that the t(11;22)(q23;q11) formed in human sperm is transcription-dependent but not meiosis-dependent.

As a conclusion, we observed the endogenous cruciform DNA foci in growing oocytes and the formation of cruciform DNA is transcription-dependent. However, which factors cause the genome instability at cruciform DNA region is still need to be investigated. Lastly, the growing mouse oocytes could be used as a model to analyze the cruciform DNA mediated genome instability and Parp1 could be used to verify or live-cell observe the cruciform DNA in growing oocytes.
